# The Treatment of Nephritic Colic*A paper giving personal experience, read before the Buffalo Medical Club, Sept. 15th, 1880.

**Published:** 1880-11

**Authors:** Charles G. Stockton


BUFFALO

### MEDICAL AND SURGICAL JOURNAL.

VOL. XX.—NOVEMBER, 1880.—No. 4.

### Original Communications.

### THE TREATMENT OF NEPHRITIC COLIC.*

BY CHARLES G. STOCKTON, M. D.

AFTER the lapse of 2,000 years, the etiology of calculous deposits is nearly as obscure as in the days of Hippocrates, and, although our knowledge of the nature of urinary calculi has greatly increased since the investigations of Wallaston, throwing much light upon the treatment, enough yet remains for discovery and research.

The pain occasioned by renal calculus differs widely both in kind and degree, depending upon the character of the concretion, the peculiarity of the patient, and a variety of other circumstances. Lodged in the pelvis of the kidney, the stone is irregular in its manifestations; at times producing no discomfort, again, a sense of oppression is felt in the region of the affected organ, varied by a dull, full, bursting pain that radiates through the sympathetic nervous system, causing anorexia, looseness of the bowels, frequent and scanty micturition, and marked irritability and peevishness. Active treatment is not here indicated.

*A paper giving personal experience, read before the Buffalo Medical Club, Sept. 15th, 1880.

Rest, with a mild diuretic, and a dose of bromide of potassium, has often tided me over such attacks. The brisk secretion of urine appears to lessen the irritability of the kidney, and copious draughts of pure or mildly alkaline water relieves the patient.

Sydenham enthusiastically advocates the use of beer "To cool off the ardent humors that remain in the kidneys and produce stone." Segalas and more recent writers re-inforce this statement. I found that beer aggravated these symptoms, as did sour wine and cider. Most writers pronounce beer and all fermented drinks injurious. Sydenham having experienced personal benefit from the frequent use of cathartics, recommends the use of manna and lemon juice. (This acted unfavorably in my own case where the stone was oxalate of lime.)

But diluents, recommended by the most ancient of medical authorities, still best subserve our purpose at this time. So much for a condition of comparative repose. It is after some unusual exertion that a sensation of uneasiness and qualmishness, is experienced, followed soon by intense pain in the lumbar and inguinal regions of the affected side; this shoots down into the corresponding testicle, which alone, or with its fellow, is violently retracted. Micturition is painful and almost continuous, the urine scanty, highly acid, of a grayish hue and mingled with blood. The gastric apparatus is greatly disturbed; there is flatulence, colic, frequent dejections, tenesmus.

The pain is best controlled by morphia and atropia, hypodermically, and the application of heat either by long continued general bath, or by rubber hot-water bags placed under the back and about the scrotum. I can not speak too highly of the hot-water bag as an epithem. It is more efficacious than the bath since it can be used continuously without fatigue to the patient.

The stone may remain in the kidney indefinitely, or it may descend the ureter into the bladder, and language fails to portray the torture that accompanies this transit. The attack is ushered in with a sudden aggravation of the symptoms before described.

The pain assumes the paroxysmal type, and, together with the attending wretchedness, is of such an overwhelming nature as to paralize the stoutest individual. Print says: "I have known nephritic colic accompanied by syncope, and in one or two cases by epilepsy,"

Owing to the peculiar formation of the ureter, narrow where it communicates with the bladder, the greatest distress is felt just before the calculus escapes into that vessel, after which the acute pains commonly cease.

Curative treatment, that calculated to dissolve and expel the concretion, in selected cases, is efficacious.

Roberts says: "For therapeutical purposes, urinary calculi may be divided into two classes, viz., those which are *soluble in alkalies*, and those which are *soluble in acids.* To the former category belong uric acid, the urates, and cystine; to the latter phosphatic and mulberry calculi. Those which are soluble in alkalies may (conceivably) be attacked by alkalizing the urine by means of certain salts administered by the month. * * * Acids can not be made to pass through the kidneys, save in insignificant proportions.

It will, * * be shown * * also, that mulberry calculi are unassailable by any solvent method hitherto proposed; so that the solvent treatment of urinary calculi resolves itself, practically, into * * attacking uric acid calculi (and their congeners) by alkalizing the urine by means of medicines administered internally * *."

These views are well grounded, but as five-sixths of all renal deposits are uric acid, we may expect a fair proportion of good results.

The solvent treatment is old. In 1739 a remedy for stone, the nostrum of Joanna Stephens, "composed of burnt egg-shells, and snails, with Alicant soap," attracted great attention in England. It fell into disrepute from being used in all cases of urolithiasis. The alkaline treatment was somewhat revived by the discoveries of Wallaston and Fourcroy, concerning the nature and composition of urinary calculi.

Potash has greater solvent powers than soda upon uric acid. Roberts advised the use of the citrate or acetate, in a solution containing one drachm (3.89) to the pint, (496.) to be given every four hours. Others recommend carbonate of potash, the bicarbonate of soda, the carbonate of lithia. Heller prefers phosphate of soda and prescribes doses of six drachms, (23.3). The treatment must be continued for weeks, or until the stone is supposed to be dissolved, always discontinuing the alkalies when the urine becomes ammoniacal.

Exercise, with diluents, sometimes succeeds in dislodging the stone, bringing it into the bladder. .

Something remains to be said of the preventive treatment and therefore of the etiology of the disorder. Atmospheric influences exert considerable power in changing the character of the urine, as all have noticed in our variable climate. Dickinson has collaborated the following table from the Registrar's Reports, showing the proportion of deaths from all kidney diseases in the various cities mentioned.

| | Cases. | Mean Temperature. |
|---|---|---|
| Aberdeen, - - - | 1 in 49 | 49—0 |
| London, - - - - | 89 | 50—3 |
| Edinburgh, - - - - | 95 | 47—2 |
| Dundee, - - - - | 107 | —— |
| Melbourne, - - - - | 110 | 57 — |
| Glasgow, - - - - | 142 | 46—9 |
| Paris, - - - - - | 266 | 52—4 |
| Bombay, - - - - | 2800 | 80—6 |
| Genoa, - - - - - | 4303 | 61 — |

One case in 49, at Aberdeen, compared with 1 in 4303, at Genoa, proves the influence of climate.

But it was not alone the cold of Aberdeen that made its average. Dr. Kane's men endured the most extreme cold without one instance of kidney affection. The same is said of Franklin and Parry.

The respiratory function is here so much exalted that the stress of excretion falls upon the lungs, and the oxydation is so active that much passes off by the lungs that in warmer climates would pass as excrementitious matter from the kidneys. The comparative immunity from kidney affections enjoyed by warm climates, may be explained on similar principles; other excreting organs than the kidneys are in a state of increased activity. A failure in the action of the skin would occasion a demand upon the liver and alimentary canal rather than upon the kidneys. In the temperate climate it appears that the kidneys sympathize and alternate with the skin more than do any other organs. So that, an exposure which in England would produce a nephritis, in India would develop hepatitis or dysentery. Materials that would be eliminated by the skin when active, are by cold forced to the kidneys, and this under unfavorable circumstances results in disturbances of those organs. These are the views of Dickinson expressed with regard to kidney diseases in general.

Ebstein, of Gottengen, says: "It was formerly thought that certain regions enjoyed immunity from calculous diseases. It was said, for instance, that the tropics were free from such affections. This is a mistake." He cites cases in India and Vera Cruz, and inclines to the belief that warm climates predispose to calculous deposits, because of the concentrated condition of the urine when the skin is active. This observation, however, is not borne out by facts.

Calculi, it is stated, depend largely upon mal-assimilation for their origin; and that besides climatic influences two other great factors are always to be considered, viz., diet and exercise. Regarding our diet—food and water—the profession has always been alert. Although in a state of ignorance regarding the more recondite vitalizing changes in digestion, we know that calculous deposits are related to disturbed assimilation.

Hippocrates says: "Men become affected with stone and kidney diseases from drinking all sorts of water."

There are some well authenticated cases in which the eating of strawberries is always followed by attacks of nephralgia, simulating nephritic colic. * * * This is a special case, but it indicates a principle.

Enormous eating should be proscribed; the meals should be small, frequently and regularly taken. Hard, particularly lime waters, should be interdicted; but the drinking in abundance of wholesome waters advised.

Upon drinking Niagara water I find the urine more acid and turbid. Drinking Bethesda, Apollinaris, or rain waters, increases and clears the urine, rendering it less acid. This is especially true of Bethesda water.

The Gettysburg katalsyne is an agreeable water, and has a favorable reputation as a remedy in renal concretions, as its constituents would lead us to suppose.

Probably the springs of Vichy provide the best waters for cases of the uric acid diathesis.

The Celestine spring is most frequented by patients with gravel, though without any apparent reason, as the spring is less alkaline than its fellows.

The Malvern water is a favorite in England. It is almost perfectly pure; so far as I know, the purest spring water on record. There is no end of good waters. I have for some time been drinking "Rosicrucian springs" water; it comes from Maine, is very agreeable, and is said to be like Apollinaris without the gas.

